# Effect of Zofenopril on regeneration of sciatic nerve crush injury in a rat model

**DOI:** 10.1186/1749-7221-4-6

**Published:** 2009-06-09

**Authors:** Ali Murat Kalender, Ali Dogan, Vedat Bakan, Huseyin Yildiz, Mehmet Ata Gokalp, Mahmut Kalender

**Affiliations:** 1 Department of Orthopedics and Traumatology, Kahramanmaras Sutcu Imam University, Medical Faculty, K. Maras, Turkey; 2 Department of Orthopedics and Traumatology, Yuzuncu Yil University, Medical Faculty, Van, Turkey; 3 Department of Pediatric Surgery, Kahramanmaras Sutcu Imam University, Medical Faculty, Kahramanmaras, Turkey; 4 Department of Anesthesiology and Reanimation, Kahramanmaras Sutcu Imam University, Medical Faculty, Kahramanmaras, Turkey; 5 Gaziantep Medical Center, Gaziantep, Turkey

## Abstract

**Background:**

Zofenopril is an antioxidant agent which has been shown to have beneficial effects in hypertension and heart failure. The aim of this study was to test the effects of Zofenopril on nerve regeneration and scarring in a rat model of peripheral nerve crush injury.

**Methods:**

Twenty-one adult Sprague-Dawley rats underwent a surgical procedure involving right sciatic nerve crush injury. 15 mg/kg Zofenopril was administered orally to seven rats in group Z for seven days. Seven rats in group S received saline orally for seven days. Seven rats in the control group C received no drug after crush injury. Fourteenth and 42nd days after injury, functional and electromyography assessments of nerves were performed. Functional recovery was analyzed using a walking track assessment, and quantified using the sciatic functional index (SFI). After these evaluations, all rats were sacrificed and microscopic evaluations were performed.

**Results:**

The Sciatic functional Index (SFI) in group Z on 14^th ^day is different significantly from group S and group C (p = 0.037). But on 42^nd ^day there was no difference between groups (p = 0.278). The statistical analyses of electromyelographic (EMG) studies showed that the latency in group Z is significantly different from group S (p = 0.006) and group C (p = 0.045). But on 42^nd ^day there was no difference between groups like SFI (p = 0.147). The amplitude was evaluated better in group Z than others (p < 0.05). In microscopic evaluation, we observed the highest number of nerve regeneration in the group Z and the lowest in the group C. But it was not significant statistically.

**Conclusion:**

Our results demonstrate that Zofenopril promotes the regeneration of peripheral nerve injuries in rat models.

## Introduction

Nerve injuries in extremity surgery occur usually by crush or tension type rather than incision or rupture. Orthopedic surgeons strive these type problems while treating long bone fracture and some times after surgical operations. Demyelinization and remyelinization, axonal degeneration and regeneration, focal, multifocal or diffuse nerve fiber loss and endoneural edema may be encountered due to crush injury [1-3]. It is also known that free oxygen radicals increase and cause tissue damage due to the tissue destruction after the injury [3,4].

There is an extensive degeneration of the distal segment, known as Wallerian Degeneration after an axonal lesion [1]. The proximal stump that is connected to the cell body can regenerate to reinnervate the target organs especially in the peripheral nervous system. Although this process is often facilitated by a permissive environment in the periphery, some factors can impede normal return to function, such as the distance from injury site, metabolic disturbances, age and type of lesion [5-8]. Experimentally, a lot of medications were used in rat crush injury models such as steroids, nonsteroidal anti-inflammatory drugs and vitamins [9-11]. Some antioxidants such as Acetyl-L carnitine (ALCAR), FK506, polyethylene glycol (PEG) are used experimentally in treatment of nerve crush injuries [12-14].

Angiotensin-converting enzyme (ACE) inhibitors are drugs with different structures and activities used to treat heart failure and hypertension [15]. Zofenopril and captopril are the only ACE inhibitors with sulphydryl groups (SH) and consequent potential antioxidant activity [16]. This activity may contribute to the notable cardio- and endothelium protective effects of Zofenopril [17].

In this study, we have evaluated the effect of Zofenopril on functional recovery following sciatic nerve crush injury in rats.

## Methods

The experimental protocols have been reviewed and approved by our University Animal Care and Ethic Committee. All efforts were made to minimize the number of animals used and their distress. 21 adult Sprague-Dawley rats weighing 250–275 g underwent unilateral (right) sciatic nerve crush. Test animals in group Z received Zofenopril (15 mg/kg/day for 7 days) (n = 7), group S received normal saline for 7 days following surgery (n = 7), and group C control animals (n = 7). The animals were kept in standard room conditions and fed with standard rat diet and water ad libitum.

All of the operations were performed under the microscope by same surgeon. The right lateral thigh was operated, after shaving and preparing the skin with 10% povidone iodine. The sciatic nerve was exposed by opening the fascial plane between the gluteal and femoral musculature via a longitudinal incision. Under kethamine anesthesia, the sciatic nerve of 21 rats was exposed at mid-thigh level and either crushed for 30 seconds with a pair of jewelers forceps (n = 16). The wound was sutured in layers and the animals were allowed to recover.

At 2^nd ^and 6^th ^weeks, all animals were evaluated for sciatic functional index (SFI) by walking tract analysis (WTA) and electromyelography (EMG).

At 6 weeks after the evaluation, in order to confirm the nerve recovery, all animals were euthanatized by cervical dislocation. A 10-mm-long sample of the right sciatic nerve segment centered to the lesion was removed, fixed, and prepared for light and electron microscopic examination. From seven random of these rats, a 10-mm-long sample of the left sciatic nerve segment without any injury was removed, fixed, and prepared for histopathological examination and histomorphometry of myelinated nerve fibers.

### Walking tract analysis

Functional recovery was analyzed using a WTA, and quantified using the sciatic functional index (SFI) [18]. Rats were tested at 14^th ^and 42^nd ^days after injury. Paw-prints were recorded by painting the hind paws with black ink and having them walk along an 8 × 80 cm corridor, lined with white paper. The paw-prints were collected. Paw length and toe spread were measured. SFI was calculated according to the following Medinacelli formula [19]:

Where ETS is the experimental toe spread, NTS the normal toe spread, EPL the experimental paw length, and NPL is the normal paw length.

### Motor nerve conduction velocity (MNCV)

At the 14^th ^and 42^nd ^days after crush injury, the MNCV studies were performed under general anesthesia, and were carried out with a Neuromatic 2000 M/C Neuro-Myograph (Dantec Elektronic Medicinsk Og Videnskabeligt Maleudstyr A/S, Skovlunde, Denmark). The sciatic nerve was percutaneously stimulated with supramaximal stimulus intensity through monopolar needle electrodes, proximal to the injury site at the level of the sciatic notch, and distal to the lesion at the level of the ankle. Square wave stimulus pulses of 500 μsec in duration were delivered at 1 Hz. Recorded signals were amplified with an alternating current-coupled preamplifier with filters at 1 Hz and 10 KHz. The latency of the evoked muscle action potentials were recorded from the intrinsic foot muscles with surface electrodes. Finally, the distance between the two sets of stimulating electrodes was measured on the skin with a ruler to the nearest 1 mm, and the conduction velocity was calculated. Both experimental (right) and normal (left) nerves were measured.

### Morphological analysis

The crushed sciatic nerves were immersed immediately just after sacrification in a drop of fixation solution, containing freshly prepared, ice cold 4% paraformaldehyde for an hour. Then, they were incubated at 0.5% saccharose solution in PBS buffer overnight. and embedded on cryomatrix (Shandon). 10 μm thick transverse frozen sections were cut using a cryomicrotome (Leica, CM1900). Sections were kept in a humidified chamber with wet gauze. 10 μL blocks solution, including 0.1% triton-X, was added to each section. Panaxonal marker NE 14 (anti-nfh antibody) is used for immunhistochemical staining as primary and anti Mouse IgG 488 antibody as secondary. Macroscopical nerve evaluation has been performed according to regenerated axon number by immunoflourescent technique. The sections were analyzed using confocal microscope (Zeiss LSM 510 Meta). Crushed, proximal and distal to crushed area of the sciatic nerve were sectioned two times and the averages used for evaluation. They were compared for immunoreactivity with image analysis. Staining intensity of the crushed, proximal, distal regions were recorded as percentile. Each group of experimental rats analyzed statistically.

### Statistical Analysis

The data were expressed as means ± SD. Distributions of the data of the groups were assessed with one-sample Kolmogorov-Smirnov Z test and were found normal (P > 0.05). One-way analysis of variance (ANOVA) was performed on the data to examine differences among groups. If a significant group effect was found, a Tukey HSD test was used to identify the location of differences between groups. A p value less than 0.05 was statistically significant. Independent Student t test was used to compare EMG values of intact extremity and operated extremity.

## Results

### Walking-track analysis

**Figure 1:**
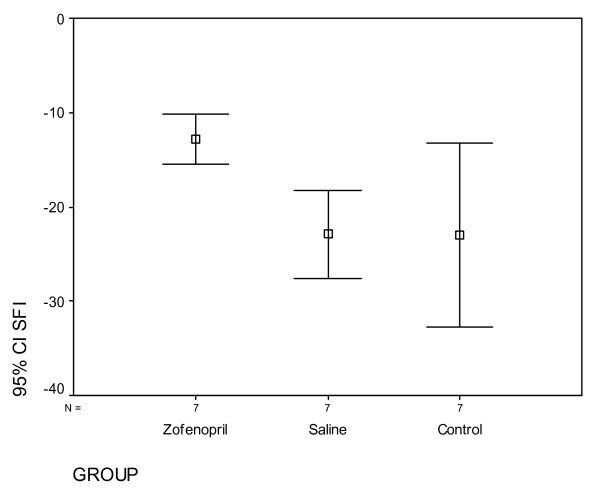
**Sciatic function index (SFI) results for 2^nd ^week, CI. Confidence intervale**.

The SFI was greatly decreased for both control and experimental groups 14 days post-injury, and began showing signs of recovery on day 42^nd^. The SFI values of group Z and S (p = 0.037) and C (p = 0.034) were significantly higher degree in the second week (Figure 1). At sixth week SFI values were close to each other in all groups. There was not a statistical difference between groups (p = 0.278). SFI values for 2^nd ^and 6^th ^weeks are given in Table 1 and Table 2.

**Table 1:** EMG results for 2^nd ^week, *: Group Z is significantly different.

	Zofenopril (Group Z) (n = 7)	Saline (Group S) (n = 7)	Control (Group C) (n = 7)	P
SFI (mean ± sd, range)	-12.84 ± 2.86 [(-16.92) – (-8.62)]	-22.88 ± 5.03* [(-32.88)-(-17.55)]	-23.02 ± 10.53* [(-39.51)-(-12.52)]	0.019
Latency (msec, mean ± sd, range)	1.51 ± 0.28 (1.10–1.90)	2.08 ± 0.23* (1.80–2.50)	1.90 ± 0.36* (1.40–2.40)	0.007
Amplitude (mV, mean ± sd, range)	8.77 ± 2.08 (5.70–11.50)	5.12 ± 1.39* (3.60–7.90)	4.94 ± 1.34* (3.80–7.50)	< 0.001

**Table 2:** EMG results for 6^th ^week,

	Zofenopril (Group Z) (n = 7)	Saline (Group S) (n = 7)	Control (Group C) (n = 7)	P
SFI (mean ± sd, range)	-7.19 ± 2.38 [(-12.28) – (-5.67)]	-9.50 ± 3.35 [(-14.11)-(-5.53)]	-12.20 ± 8.90 [(-31.74)-(-5.67)]	0.278
Latency (msec, mean ± sd, range)	1.33 ± 0.23 (1.00–1.60)	1.70 ± 0.31 (1.40–2.20)	1.60 ± 0.46 (1.20–2.40)	0.147
Amplitude (mV, mean ± sd, range)	12.61 ± 2.69 (9.10–16.00)	11.03 ± 3.52 (6.30–17.00)	10.31 ± 2.88 (6.90–14.40)	0.374

The EMG studies of the Subjects on the 14^th ^day showed that right sciatic nerve has a severe injury according to left (intact) side that is statistically different (paired t test) (T = -3.31 P = 0.016).

**Figure 2:**
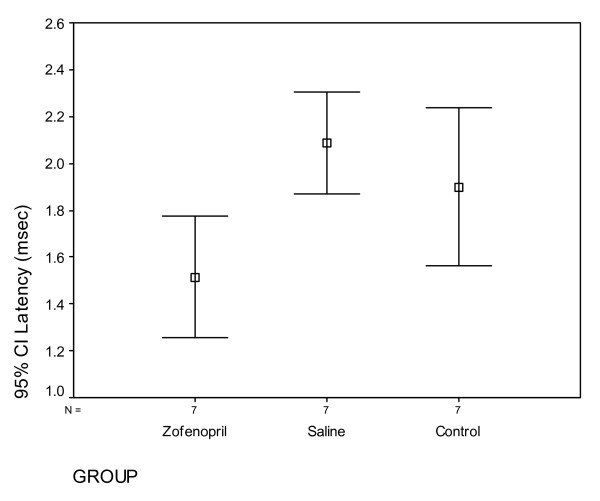
**EMG results for 2^nd ^week, (latency), CI. Confidence intervale**.

**Figure 3:**
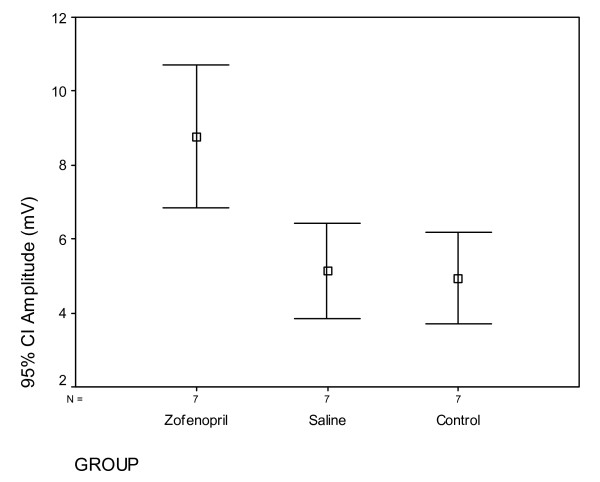
**EMG results for 2^nd ^week, (amplitude), CI. Confidence intervale**.

The EMG measurement of rats in the second week for the latency significant degree between the groups are different (p = 0.007). The latency in the 2^nd ^week of the group Z was significantly lower than group S (p = 0.006) and C (p = 0,045) (Figure 2). But this difference disappeared in the 6^th ^week (p = 0,147). EMG results for 2nd and 6^th ^weeks are given in Table 1 and 2. The amplitude values are examined, similar to the latency, there was a significant difference between the groups (p < 0,001) at 2^nd ^week, but not on the 6^th ^week (p = 0,374) (Figure 3).

### Morphological analysis results

**Figure 4:**
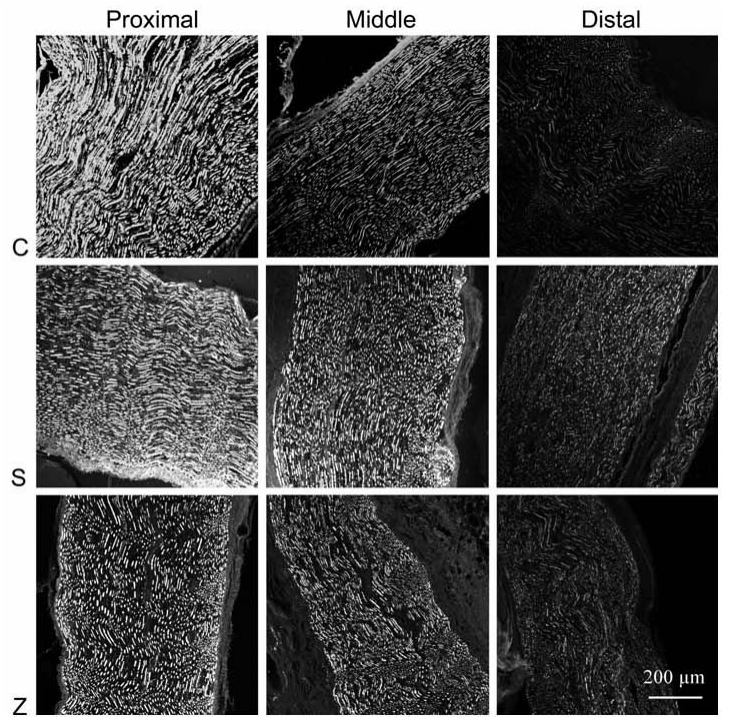
**NFH immunoreactivity in the sections of proximal, middle (crush site) and distal parts of the sciatic nerves from animals in control (C), saline (S) and zofenopril (Z) groups**. The lowest regenerated fibril number estimated in group C, and highest in group Z.

In all groups, lesion area, the proximal and distal parts of the lesion were estimated microscopically. The number of the fibrils found decreased in the distal to lesion nerve in all groups (Figure 4). The lowest regenerated fibril number estimated in group C, and highest in group Z.

## Discussion

Severe anatomical and functional disorders can be seen after peripheral nerve injury. This type of injury frequency is increasing with technology in industrialized societies. Nerve injuries in extremity represent usually by crush or tension type rather than incision or rupture in surgery or trauma. Spontaneous regeneration through the distal nerve stump with good functional return can be expected after this type of injury [20,21]. This type of nerve injuries are treated pharmacological agents instead of surgery.

For this purpose, many pharmacological agents are tried experimentally and successful results were reported [9-14]. However, these studies did not go beyond the experimental studies. The healing process after nerve injury is reduced mainly free oxygen radicals rather than inflammation and edema [2]. Therefore, in recent years many researchers started to stand on the antioxidant mechanism. Antioxidant materials contribute nerve regeneration via free oxygen radicals scavenging effect [22]. Antioxidant enzymes such as superoxide dismutase and catalase and GSH-Px are found in mammalian organisms and protect cells from toxic effects of free radicals. While free radicals production, lipid peroxidation develops on cell membrane and this can lead to final cell death. The protective antioxidant enzyme activity increases in response to free radical formation. There are many experimental studies available showing free radicals production and importance of lipid peroxidation on cell membrane injury in nervous system injuries. Free radicals induced traumatic cell damage is basic mechanism of cell death. Nevertheless, catalase and GSH-Px traumatic damage such as the FOR cleaners provide partial improvement. [23]

Studies using the photo-oxidation of riboflavin sensitized by dianisidine to generate active oxygen species have clearly defined the remarkable difference in the antioxidant action of SH-containing compared with non-SH-containing, ACE inhibitors [24]. The SH-containing, ACE inhibitors zofenopril, captopril, epicaptopril (the stereoisomer of captopril, which is devoid of ACE inhibitory properties) and fentiapril were found to be effective scavengers of non-superoxide free radicals, while four non-SH-containing ACE inhibitors were inactive. The protective effects from free radical-induced cell damage of SH-containing ACE inhibitors have also been assessed in cultured endothelial cells exposed to a superoxide anion and hydroxyl radical generating system [25]. Pre-incubation of the cells with captopril, epicaptopril or zofenopril produced a concentration dependent (10 – 200 μM) inhibition of malonyldialdehyde formation. Both loss of cell viability and membrane blebbing were reduced by SH-containing ACE inhibitors at concentrations as low as 10 μM. In contrast, lisinopril and enalaprilat were ineffective at concentrations up to 200 μM.

Because of known antioxidant and free oxygen radicals scavenging effect of Zofenopril; it is used in experimental studies on ischemia-reperfusion damages in brain, kidney, heart and liver tissue [26,27].

It has higher lipophilic effect than other ACE inhibitors with the long-term tissue penetration features tissue. Thus the long duration of effect is provided. In this way, and vascular tissue ACE myocardium and other drugs inhibition effects last much longer and has been shown to be effective [16].

Sunderland second-degree injury or axonotmesis means a breakdown of the axon and distal Wallerian degeneration but keeping of the continuity of the endoneural sheath. Spontaneous regeneration through the distal nerve stump with good functional return can be expected after this type of injury [20,21]. As the restored pattern of innervations is identical to the original, the study of this nerve lesion provides a good model for establishing the ontogeny of functional nerve recovery.

Electrophysiological, morphological and histologic studies were used for evaluation of experimental peripheric nerve regeneration [1-5]. But none of them was enough to determine the nerve recovery. Medinacelli at al. reported walking gait analysis for rat sciatic nerve. Later this method is modified and named as sciatic functional index [3].

The SFI increased and normal values were achieved at week 7 after sciatic nerve injury. Several authors reported nearly same results whose studies have also shown normal walking patterns only after the first month of post crush [28,29]. In contrast to these experiments, some authors reported a full recovery at the third and fourth weeks [30]. The difference in the rate of motor functional recovery may relate to the pathophysiologic response of peripheral nerves to the magnitude of different crushing loads [31].

In this study, the SFI in Zofenopril group was significantly higher than other groups in 2^nd ^week. We believe that this medication accelerates nerve crush injury healing in rats. Our findings in SFI and EMG studies in 2^nd ^week support this improvement. In the second week after injury and the EMG test results done in six weeks on the morphological analysis results support these findings.

## Conclusion

As a result, Zofenopril has been found effective in promoting nerve regeneration in sciatic nerve crush injury rat model. These molecules can be used also for the human injured nerve but additional work is needed.

## Competing interests

The authors declare that they have no competing interests.

## Authors' contributions

AMK designed the study and performed experimental operations. AD and VB performed statistical analyses. HY and MAG had performed final operations and specimen collection of this experimental study. MK had performed linguistic and technical corrections. All authors read and approved the final manuscript.
